# Dose-Dependent Inhibitory Effect of Probiotic *Lactobacillus plantarum* on *Streptococcus mutans*-*Candida albicans* Cross-Kingdom Microorganisms

**DOI:** 10.3390/pathogens12060848

**Published:** 2023-06-20

**Authors:** Jianhang Bao, Xinyan Huang, Yan Zeng, Tong Tong Wu, Xingyi Lu, Gina Meng, Yanfang Ren, Jin Xiao

**Affiliations:** 1 Eastman Institute for Oral Health, University of Rochester Medical Center, Rochester, NY 14642, USA; jianhang_bao@urmc.rochester.edu (J.B.);; 2 School of Stomatology, Henan University, Zhengzhou 450046, China; 3 Department of Biostatistics and Computational Biology, University of Rochester Medical Center, Rochester, NY 14642, USA; 4 School of Arts and Science, University of Rochester, Rochester, NY 14627, USA

**Keywords:** *Lactobacillus plantarum*, probiotic, cross-kingdom interaction, fungi, *Streptococcus mutans*, *Candida albicans*

## Abstract

Dental caries is one of the most common chronic diseases worldwide. *Streptococcus mutans* and *Candida albicans* are two major pathogens associated with dental caries. Several recent studies revealed that *Lactobacillus plantarum* inhibits *S. mutans* and *C. albicans* in biofilms and in a rodent model of dental caries. The aim of this study was to investigate the dose-dependent effect of *L. plantarum* against *S. mutans* and *C. albicans* in a planktonic model that simulated a high-caries-risk clinical condition. Mono-, dual-, and multi-species models were utilized, with five doses of *L. plantarum* (ranging from 1.0 × 10^4^ to 1.0 × 10^8^ CFU/mL). Real-time PCR was used to assess the expression of the virulence genes of *C. albicans* and *S. mutans* and the genes of *L. plantarum*. Student’s *t*-tests and one-way ANOVA, followed by post hoc tests, were employed to compare the cell viability and gene expression among groups. A dose-dependent inhibition on *C. albicans* and *S. mutans* was observed with increased dosages of *L. plantarum*. *L. plantarum* at 10^8^ CFU/mL demonstrated the highest antibacterial and antifungal inhibitory effect in the dual- and multi-species models. Specifically, at 20 h, the growth of *C. albicans* and *S. mutans* was suppressed by 1.5 and 5 logs, respectively (*p* < 0.05). The antifungal and antibacterial effects were attenuated in lower doses of *L. plantarum* (10^4^–10^7^ CFU/mL). The expression of *C. albicans HWP1* and *ECE 1* genes and *S. mutans lacC* and *lacG* genes were significantly downregulated with an added 10^8^ CFU/mL of *L. plantarum* (*p* < 0.05). The addition of 10^8^ CFU/mL *L. plantarum* further inhibited the hyphae or pseudohyphae formation of *C. albicans*. In summary, *L. plantarum* demonstrated dose-dependent antifungal and antibacterial effects against *C. albicans* and *S. mutans*. *L. plantarum* emerged as a promising candidate for the creation of novel antimicrobial probiotic products targeting dental caries prevention. Further research is warranted to identify the functional metabolites produced by *L. plantarum* at different dosages when interacting with *C. albicans* and *S. mutans*.

## 1. Introduction

Dental caries is one of the most common chronic diseases worldwide [1]. It is a microbial, sugar-driven, complex, dynamic disease characterized by the phasic demineralization of dental hard tissues [2]. Caries development is associated with fermentable sugar, host susceptibility, cariogenic microflora, and other environmental factors [3,4]. *Streptococcus mutans* is considered as the main culprit for dental caries, as it is acidogenic and aciduric and has the capability of assembling an extracellular matrix that is essential for dental biofilms (plaque) formation [5].

*Candida albicans*, an opportunistic fungal pathogen, is considered as the main cause of oral candidiasis [6]. A recent systematic review revealed that children with oral *C. albicans* are more likely to have early childhood caries than children who do not have oral *C. albicans* [7]. Rodent models further demonstrated that *C. albicans* can be cariogenic [8] and play a critical role in root caries [9]. In mixed-species biofilms, *C. albicans* promoted *S. mutans* accumulation and extracellular matrix formation [10,11,12,13]. In vitro, *S. mutans* and *C. albicans*’s symbiotic relationship increased the virulence of plaque biofilms and resulted in more severe caries on smooth surfaces [11].

Dental caries is traditionally controlled by mechanical non-specific oral biofilm removals, such as tooth brushing and dental flossing. In addition, chlorhexidine is often used as an antibacterial and antifungal agent in caries prophylaxis [14]. Recent studies adopted an antifungal approach for caries risk reduction [15,16]. However, the long-term efficacy of such chemical intervention remains unclear. An ecological approach for caries prevention has emerged and is considered a more desirable strategy, compared to chemical interventions [17].

Probiotics are live microorganisms that benefit the host’s health when given in sufficient amounts [18]. Many probiotics are sold as dietary supplements. Some foods are rich in probiotics, such as Greek yogurt, kefir, and kimchi. Probiotic bacteria have been used to preserve health quality for decades. *Lactobacillus* is one of the most commonly used probiotics in commercial products [19]. Of particular interest is the use of probiotic *Lactobacillus* in caries prevention and treatment. Probiotic lactobacilli can produce lactic acid, peroxide, and bacteriocin, which inhibit the growth of potential pathogens [20].

*L. plantarum* is a facultative heterofermentative Gram-positive bacterium that is frequently used to ferment dairy products including cheese, kefir, and fermented meats and beverages [21]. According to a recent study, children without *C. albicans* infection had dental plaque *L. plantarum* levels that were three times greater than those with *C. albicans* infection [22]. This study suggests the potential of *L. plantarum* as an antifungal and antibacterial probiotic. Several recent studies revealed that *L. plantarum* inhibits the growth of *S. mutans* and *C. albicans* in biofilms and in rodents [23,24,25,26,27,28,29,30,31]. However, the optimal dosage of *L. plantarum* for the inhibition of these microorganisms has yet to be determined. The aim of this experiment in vitro is to assess the effect of various doses of *L. plantarum* against *S. mutans* and *C. albicans* in planktonic models that simulated high-caries-risk clinical conditions. This study expands our understanding of the dosage-dependent nature of the antimicrobial and antifungal aspects of *L. plantarum*, using the dual-species model comprising *S. mutans* and *C. albicans*, and provides insights into the development of novel antimicrobial and antifungal probiotic products in the context of, but not limited to, dental caries prevention.

## 2. Materials and Methods

### 2.1. Bacterial Strains and Starter Preparation

*C. albicans SC5314*, *S. mutans UA159*, *L. plantarum ATCC 14917* were used in this study. *C. albicans*, *S. mutans*, and *L. plantarum* that were recovered from frozen stock were added into YPD agar (BD Difco™, San Jose, CA, USA, 242720), blood agar (TSA with sheep blood, Thermo Scientifific™, Waltham, MA, USA, R01202), and MRS agar (BD Difco™, 288210), respectively, and grown for 48 h. Next, 3-5 colonies were incubated in 10 mL of broth overnight (5% CO_2_, 37 °C). *C. albicans*, *S. mutans*, and *L. plantarum* were grown overnight in YPD broth (BD Difco™, 242820), TSBYE broth (3% Tryptic Soy, 0.5% Yeast Extract Broth, BD Bacto™ 286220 and Gibco™ 212750) with 1% glucose, and MRS broth (BD Difco™, 288130), respectively. To reach the mid-exponential phase with desirable optical density, 0.5 mL of the overnight starter were transferred into individual glass tubes with broth and cultured for about 4 h on the second day. Based on the standard curves for these three strains, when morning starters reached the target OD, the represented concentration of *C. albicans* was 10^6^ CFU/mL, *S. mutans* was 10^8^ CFU/mL, and *L. plantarum* was 10^9^ CFU/mL. The morning starters were then serial dilution into starting concentration for the planktonic models described below [28,32].

### 2.2. Planktonic Model

The starting concentration for microorganisms was 10^3^ CFU/mL for *C. albicans*, 10^5^ CFU/mL for *S. mutans*, and 10^4^-10^8^ CFU/mL for *L. plantarum*. *C. albicans* (10^3^ CFU/mL) and *S. mutans* (10^5^ CFU/mL) were used in this model to mimic a high-caries-risk clinical condition [33]. The highest inoculation level of *L. plantarum* (10^8^ CFU/mL) corresponded to the lower dosage of probiotics utilized in commercial probiotic products (10^9^–10^12^ CFU/mL as a single dosage).

Mono-species, dual-species, and multi-species models were used to assess the interaction amongst *C. albicans*, *S. mutans*, and different doses of *L. plantarum* (10^4^–10^8^ CFU/mL). The experimental design of the study is shown as a schematic in Figure S1. The planktonic models consist of three classes: mono-species, dual-species, and multi-species. For the mono-species models, *C. albicans*, *S. mutans,* or one of the five dosages of *L. plantarum* (10^4^-10^8^ CFU/mL) were incubated in 10 mL of TSBYE broth with 1% glucose for 20 h (5% CO2, 37°C). For the dual-species models, either *C. albicans* or *S. mutans* was co-cultured with one of the various doses of *L. plantarum* (10^4^–10^8^ CFU/mL) for 20 h under the same conditions. For the multi-species models, *C. albicans*, *S. mutans*, and one of the different doses of *L. plantarum* (10^4^–10^8^ CFU/mL) were cultivated for 20 h under the same circumstances. The colony-forming unit per milliliter (CFU/mL) and pH value were measured at selected time points for each model.

Inhibition of *C. albicans* hyphae/pseudohyphae formation was evaluated by observing the 20 h culture medium under a light microscope (Olympus BX43, 214, Tokyo, Japan) with a 100X oil objective (Olympus UPlanFL N 100X, Tokyo, Japan). Then, 20 µL of culture medium was placed on the glass slide and viewed without staining.

### 2.3. Quantitative Real-Time Reverse Transcription Polymerase Chain Reaction (qRT-PCR)

Quantitative real-time reverse transcription polymerase chain reaction assay (qRT-PCR) was conducted to validate particular genes related to *S. mutans*, *C. albicans*, and *L. plantarum* virulence factors or viability. The specific genes of interest and primers used in this study are shown in Tables S1 and S2. First, RNAs were collected and extracted from 4 mL culture media at 6 and 20 h. Then, 0.2 μg of purified RNA were used to synthesize complementary DNAs (cDNAs) with an iScript cDNA Synthesis Kit (Bio-Rad Laboratories, Inc., Hercules, CA, USA)). Negative controls and the resultant cDNA were quantitatively amplified using Applied Biosystems™ PowerTrack™ SYBR Green Master Mix and a QuantStudio™ 3 Real-Time PCR System (Thermo Fisher Scientific, Wilmington, DE, USA). Each 20 μL of PCR reaction comprised cDNA template, 10 μM of each primer, and 2× SYBR-Green mix (SYBR-Green and Taq DNA Polymerase). Three replicates were set up, and relative gene expression was determined using the comparative ΔΔCt method [34]. Unique core genes of *C. albicans*, *S. mutans*, and *L. plantarum* were utilized as the housekeeping genes for gene expression comparisons: *ACT1* for *C. albicans*, *gyrA* for *S. mutans* genes, and *ropB* for *L. plantarum*.

### 2.4. Statistical Analysis

To compare the abundance of *S. mutans*, *C. albicans*, and *L. plantarum* in planktonic models, the CFU/mL values were first converted into natural log values before analysis. Zero values were retained as zero. Normality tests were initially conducted for measurements of pH value, converted CFU/mL value, and 2^−ΔΔCT^ at selected time points. When data were normally distributed, the difference between groups were examined using Student’s *t*-test for two groups and one-way ANOVA followed by a post hoc test for more than two groups. Nevertheless, when data were not normally distributed, the Mann–Whitney U test was used to compare the results of the two groups, whereas the Kruskal–Wallis test was used to compare the results for more than two groups. Tests of statistical significance were two-sided with a significance level of *p* < 0.05. All analyses were performed in SPSS Version 24 (SPSS Statistics for Windows, Version 24.0; IBM, Armonk, NY, USA).

## 3. Results

### 3.1. Dose-Dependent Inhibition of L. plantarum on C. albicans in Dual- and Multi-Species Conditions

**Figure 1:**
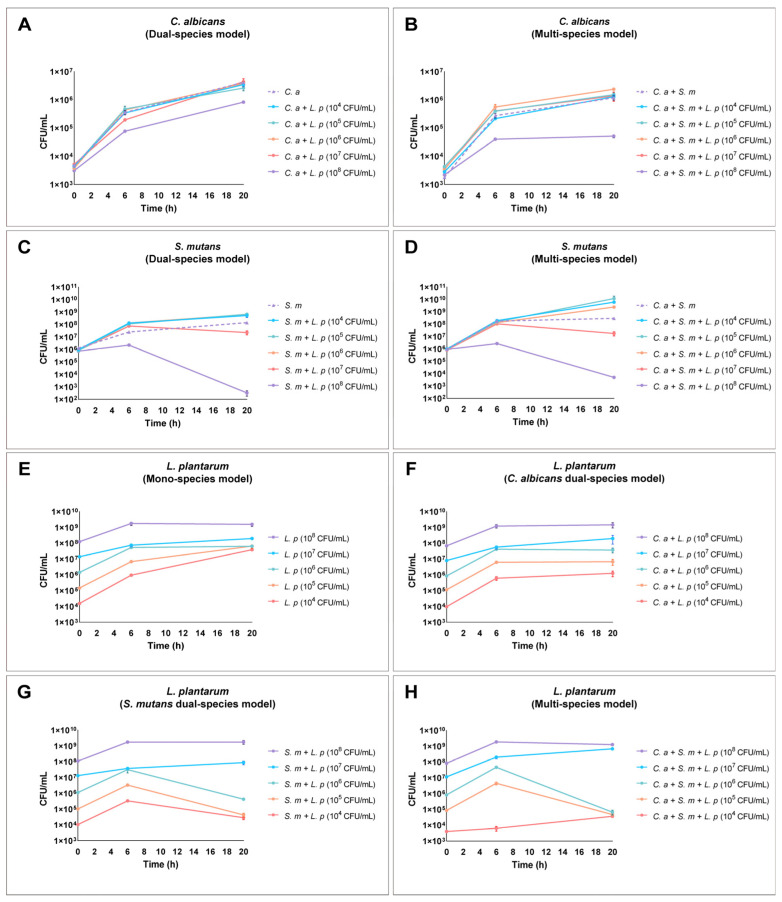
Dynamic changes in the viable cells of *C. albicans*, *S. mutans*, and *L. plantarum* in mono-, dual-, and multi-species models. (**A**,**B**) *C. albicans* viable cells in dual- and multi-species conditions. (**C**,**D**) *S. mutans* viable cells in dual- and multi-species conditions. (**E**) *L. plantarum* viable cells in mono-species condition. (**F**) *L. plantarum* viable cells in *C. albicans* presence dual-species condition. (**G**) *L. plantarum* viable cells in *S. mutans* presence dual-species condition. (**H**) *L. plantarum* viable cells in multi-species condition. Overall, dose-dependent antimicrobial and antifungal effects were seen for *L. plantarum*; 10^8^ CFU/mL of *L. plantarum* showed inhibition on the growth of *C. albicans* and *S. mutans*. The dotted line represents the control groups in each model.

Compared to individually grown *C. albicans*, 10^8^ CFU/mL of *L. plantarum* significantly reduced the growth of *C. albicans* by 1 log at 6 and 20 h (Figure 1A). Furthermore, 10^8^ CFU/mL of *L. plantarum* had a stronger inhibition of *C. albicans* in the multi-species condition when *S. mutans* was included; the growth of *C. albicans* was inhibited by 1.5 logs at 20 h (*p* < 0.05) (Figure 1B).

### 3.2. Dose-Dependent Inhibition of L. plantarum on S. mutans in Dual- and Multi-Species Conditions

Compared to *S. mutans* that was grown alone, 10^8^ CFU/mL of *L. plantarum* significantly inhibited the growth of *S. mutans* by 2 logs at 6 h and 5 logs at 20 h in the dual-species model. However, 10^7^ CFU/mL of *L. plantarum* only inhibited the growth of *S. mutans* by 1 log at 20 h (Figure 1C). Unexpectedly, the lower doses of *L. plantarum* (10^4^–10^6^ CFU/mL) promoted the growth of *S. mutans* in the dual-species model (Figure 1C) and in the multi-species condition when *C. albicans* was included (Figure 1D, *p* < 0.05). Concurrent with this finding, the viable cells of *L. plantarum* in 10^4^–10^6^ CFU/mL conditions were significantly reduced at 20 h (Figure 1G,H).

### 3.3. Cross-Kingdom Competition between L. plantarum, S. mutans, and C. albicans

The growth of *L. plantarum* in the mono-species condition during the initial 6 h was positively associated with the starting concentration. However, at 20 h, only the condition with an inoculation concentration at 10^8^ CFU/mL reached an ending concentration of 10^9^ CFU/mL, while the groups that had an inoculation concentration between 10^4^–10^7^ CFU/mL of *L. plantarum* only reached a final concentration of 10^8^ CFU/mL (Figure 1E).

Next, we assessed the growth of *L. plantarum* in the dual-species condition when *L. plantarum* grew with either *S. mutans* or *C. albicans* and in the multi-species condition when *L. plantarum* grew with *S. mutans* and *C. albicans*. When separately interacting with *S. mutans* and *C. albicans* (dual-species model, Figure 1F,G), the competitive capability of *L. plantarum* was significantly attenuated at the lower starting concentrations of 10^4^ and 10^5^ CFU/mL. The growth of *L. plantarum* at 10^4^–10^6^ CFU/mL was significantly impeded by the presence of *C. albicans* and *S. mutans* in the multi-species model, while the growth patterns of higher doses of *L. plantarum* at 10^7^ and 10^8^ CFU/mL were not influenced (Figure 1H).

### 3.4. Dose-Dependent Ecological Shift in Multi-Species Conditions

**Figure 2:**
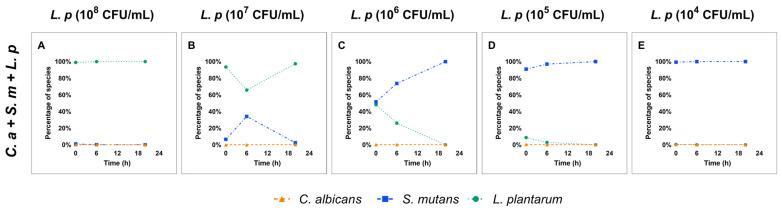
Changes in species composition in multi-species. The composition of each microorganism in multi-species condition is shown. (**A**–**E**) The composition of *C. albicans*, *S. mutans*, and *L. plantarum* in multi-species condition.

The growth competition between *C. albicans*, *S. mutans,* and *L. plantarum* in the multi-species condition is shown in Figure 2. Overall, *L. plantarum* at a dose of 10^8^ CFU/mL was able to retain its dominance when competing with *C. albicans* and *S. mutans* in the multi-species model (Figure 2A). When the starting dose of *L. plantarum* was lowered to 10^7^ CFU/mL, the level of *L. plantarum* was reduced by 37% at 6 h. However, subsequently, *L. plantarum* regained its dominance in the microbial community at 20 h (Figure 2B). When the starting dose of *L. plantarum* was lower than 10^7^ CFU/mL, *S. mutans* took over and became the dominant species at 20 h (Figure 2C–E). A similar scenario was seen in the dual-species condition when *S. mutans* was present (Figure S2F–J).

### 3.5. Dose-Dependent Effect of L. plantarum on pH Drop in Mono-, Dual-, and Multi-Species Conditions

In general, the pH of the culture medium decreased faster and to a lower value when a higher dose of *L. plantarum* was added. The pH of the culture medium decreased over time and reached a nadir at 20 h for all groups in the four models. The reduction in pH was dose-dependent in the mono-species model (10^4^–10^8^ CFU/mL *L. plantarum*). The pH value decreased to 3.7 in the 10^8^ CFU/mL *L. plantarum* group at 20 h (Figure S3A). The addition of *C. albicans* had a negligible effect on the culture medium pH, compared to *L. plantarum* alone. In other words, the trend of pH decline was nearly identical (Figure S3B). The pH in all groups significantly dropped when incubated with *S. mutans* at 20 h. For the 10^8^ CFU/mL *L. plantarum* + *S. mutans* model, the pH was found to be the lowest at 3.7 (Figure S3C). Similarly, the multi-species model (Figure S3D) showed the same pH drop trend as the *S. mutans* + *L. plantarum* dual-species model.

### 3.6. Inhibition of S. mutans Virulence Genes by L. plantarum

**Figure 3:**
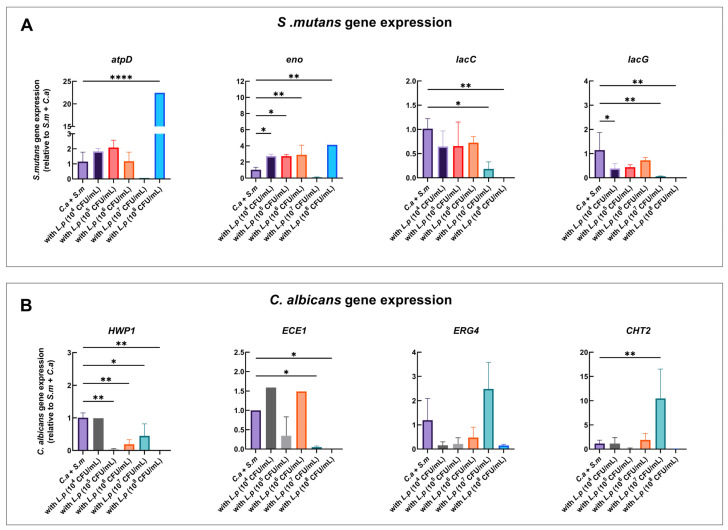
Effect of *L. plantarum* on the expression of *C. albicans* and *S. mutans* genes in multi-species model at 20 h. qRT-PCR was performed for *S. mutans* and *C. albicans* genes of interest for mixed-species culture at 20 h. *S. mutans* (**A**) and *C. albicans* (**B**) gene expression ratios are shown, and the comparison is relative to *S. mutans* and *C. albicans* dual-species. *p* values were determined by one-way ANOVA with post hoc test. * *p* < 0.05, ** *p* < 0.01, **** *p* < 0.0001.

To evaluate the differential gene expression between the control and the condition with added *L. plantarum,* qRT-PCR was conducted at 6 and 20 h (Figure S4 and Figure 3).

At 20 h, *S. mutans atpD* and *eno* genes were significantly upregulated with *L. plantarum* at 10^8^ CFU/mL in the multi-species model in comparison to the control group (*C. albicans* + *S. mutans*). *lacC* and *lacG* were significantly downregulated with *L. plantarum* at 10^7^ CFU/mL, but they could not be detected with *L. plantarum* at 10^8^ CFU/mL (Figure 3A).

### 3.7. Inhibition of C. albicans Virulence Genes by L. plantarum

For *C. albicans,* both the *HWP1* and *ECE1* genes were downregulated by 99.9% with *L. plantarum* at 10^8^ CFU/mL in the multi-species model in comparison to the control group (*C. albicans* and *S. mutans*). *HWP1* and *ECE1* was also significantly downregulated with *L. plantarum* at 10^7^ CFU/mL. *CHT2* was upregulated with *L. plantarum* at 10^7^ CFU/mL. *CHT2* expressions increased by 10.48-fold. In addition, the virulence genes *HWP1*, *ECE1*, *ERG4,* and *CHT2* were observed to be the lowest with *L. plantarum* at 10^8^ CFU/mL (Figure 3B).

### 3.8. Dose-Dependent Gene Expressions by L. plantarum in Mono-Species Model

**Figure 4:**
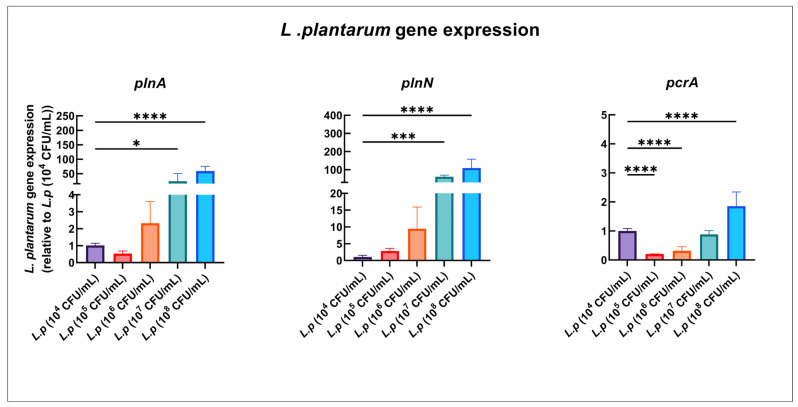
Dose-related expression of *L. plantarum* gene in mono-species model. qRT-PCR was performed for *L. plantarum* genes of interest for mono-species culture at 20 h. *L. plantarum* gene expression ratio is shown, and the comparison is relative to 10^4^ CFU/mL *L. plantarum* mono-species group. *p* values were determined by one-way ANOVA with post hoc test. * *p* < 0.05, *** *p* < 0.001, **** *p* < 0.0001.

Plantaricin are antimicrobial peptides produced by *L. plantarum*. The expression of plantaricin-related genes *plnA* and *plnN* exhibited dose-dependent effects in the *L. plantarum* mono-species model. Specifically, *plnA* and *plnN* were significantly upregulated with *L. plantarum* at 10^7^ and 10^8^ CFU/mL. For instance, *plnA* was 23.5-fold and 58.8-fold higher and *plnN* was 61.6-fold and 109.4-fold higher with *L. plantarum* at 10^7^ and 10^8^ CFU/mL, respectively, than with *L. plantarum* at 10^4^ CFU/mL (Figure 4).

### 3.9. Dose-Dependent Gene Expressions by L. plantarum in Multi-Species Model

**Figure 5:**
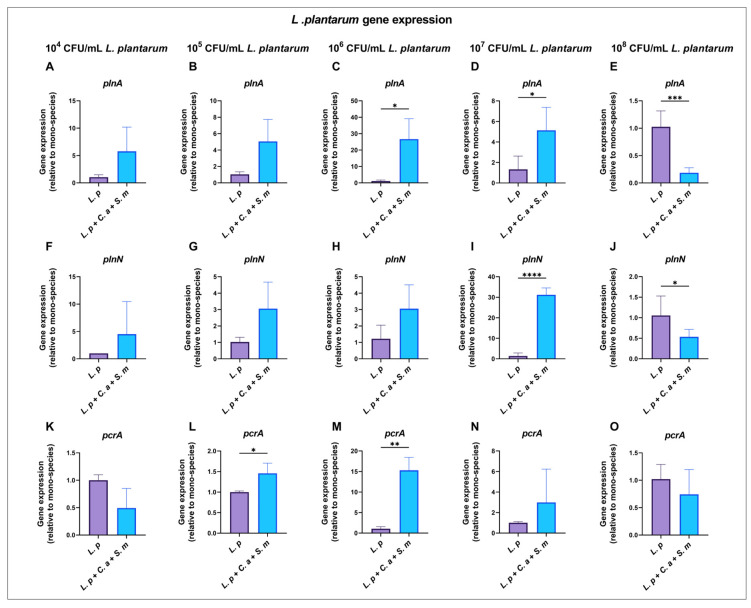
Expression of *L. plantarum* genes when interacting with *S. mutans* and *C. albicans* in multi-species model at 20 h. qRT-PCR was performed for *L. plantarum* genes of interest for mixed-species culture at 20 h. *L. plantarum* gene expression ratio is shown (**A**–**O**), and the comparison is relative to *L. plantarum* mono-species group. *p* values were determined by *t*-test. * *p* < 0.05, ** *p* < 0.01, *** *p* < 0.001, **** *p* < 0.0001.

In the multi-species model where *L. plantarum* was co-cultured with *S. mutans* and *C. albicans*, significant downregulation of *plnA* (82%) and *plnN* (49.2%) was observed, compared to the mono-species model with *L. plantarum* at 10^8^ CFU/mL (Figure 5E,J). However, the *plnA* and *plnN* genes were significantly upregulated with *L. plantarum* at 10^7^ CFU/mL in the multi-species model, compared to the *L. plantarum* mono-species model (Figure 5D,I).

In the multi-species model, the highest expressions for the *plnA* and *plnN* genes were observed with *L. plantarum* at 10^7^ CFU/mL (Figure S5).

### 3.10. Inhibition of C. albicans Hyphae/Pseudohyphae Formation

**Figure 6:**
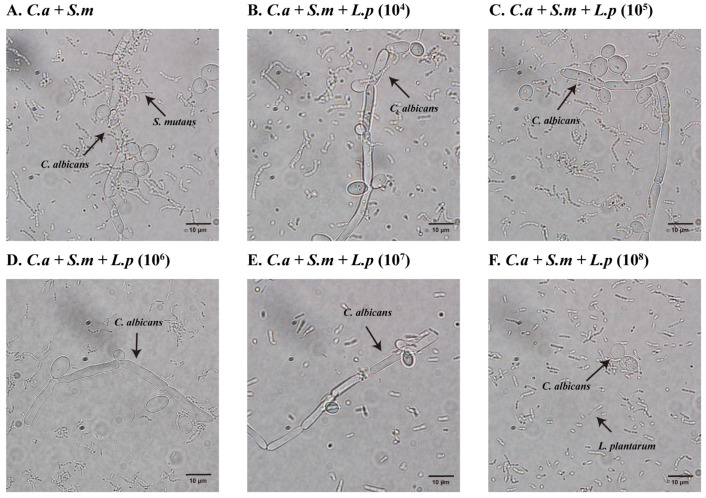
Dose-dependent inhibition of *C. albicans* hyphae formation by *L. plantarum* gene in multi-species model at × 100 magnification. (**A**) *S. mutans* and *C. albicans* grown in dual-species model at 20 h. (**B**–**F**) *S. mutans* and *C. albicans* grown with addition of various doses of *L. plantarum* at 20 h. The addition of 10^8^ CFU/mL *L. plantarum* inhibited the growth of *C. albicans* and the transition from yeast to hyphae or pseudohyphae form. These are representative images of multiple fields of view.

Inhibition of *C. albicans* hyphae or pseudohyphae formation was assessed by observing the 20 h culture medium under a light microscope. Figure 6 shows that the addition of 10^8^ CFU/mL *L. plantarum* inhibited the growth of *C. albicans* and the transition from yeast to hyphae or pseudohyphae form, compared to other groups.

## 4. Discussion

The findings of the present study indicate that the probiotic *L. plantarum* inhibited the growth of *C. albicans* and *S. mutans* in a dose-dependent manner in the dual- and multi-species planktonic models. *L. plantarum* at 10^8^ CFU/mL significantly inhibited the growth of both *C. albicans* and *S. mutans* while its own growth pattern was unaffected. *L. plantarum* at doses lower than 10^7^ CFU/mL had no significant effect on the growth of *C. albicans*, but *L. plantarum* promoted the growth of *S. mutans*.

One possible explanation for the effects of *L. plantarum* against *C. albicans* and *S. mutans* is its ability to produce organic acids. *L. plantarum* strains have the capacity to synthesize diverse organic acids, which were reported in a previous study to exhibit antimicrobial and antifungal properties [35,36]. Lactic acid and acetic acid are the two primary organic acids produced by these strains [35,37].

*C. albicans* exhibits acidogenic and aciduric properties [38] with the ability to produce acids in low pH environments, albeit less efficiently than *lactobacilli* [39]. The inhibitory effect of *L. plantarum* on *C. albicans* is consistent with an earlier report [28], but the current study shows that such an effect is only evident with *L. plantarum* at doses higher than 10^7^ CFU/mL. Though the medium pH only dropped to a level below 4.0 with *L. plantarum* at 10^8^ CFU/mL in the multi-species model, the pH levels could not explain the antifungal effect, as *C. albicans* could thrive in pH 3.0 [40]. One possible explanation is that *L. plantarum* produces acetic acid [37], which is cytotoxic against *C albicans* [36].

*L. plantarum* produces lactic acid that is the primary end-product of carbohydrate fermentation [41]. A recent study indicated that lactic acid plays a dominant role in the *L. plantarum* cell-free supernatant against *S. mutans* [42]. In addition to organic acids, *L. plantarum* can produce the antimicrobial substance plantaricin. Plantaricin (*pln*) loci are associated with plantaricin production. Each locus has around 25 genes covering a length of DNA, grouped into 5–6 operons [43]. The regulatory operons (*plnABCD*) involved in plantaricin production encode different proteins. The *plnA* gene encodes inducing peptides that regulate the transcription of the other operons [44]. In addition, the *plnN* gene encodes a putative prebacteriocin. An increased expression of genes involved in plantaricin production was observed in low pH conditions [27]. We found that the expressions of *plnA* and *plnN* genes increased with increasing doses of *L. plantarum* in the mono-species model. These genes were downregulated in the multi-species model with *L. plantarum* at 10^8^ CFU/mL. A likely explanation is that that quantity of viable cells of *S. mutans* became very low in this model, and the needs for *L. plantarum* to produce plantaricin had decreased.

The genes associated with the virulence of *C. albicans* were altered in the dual- and multi-species models containing *L. plantarum*. Both the *HWP1* and *ECE1* genes were downregulated at 20 h with *L. plantarum* at 10^8^ CFU/mL. The *HWP1* gene encoded the hyphal wall protein 1 [45], and the *ECE1* gene encoded the extent of cell elongation 1 [46]. The *HWP1* gene encoded the hyphal wall protein 1 that works as an adhesin and is critical for the integrity of cell-to-cell adhesions in biofilms [47]. *HWP1*-deficient strains of *C. albicans* were less capable of inducing systemic candidiasis in mice and were unable to form persistent attachments to human epithelial cells [47,48]. In addition, *ECE1* was tightly associated with yeast-to-hyphae transition [49]. Hyphal growth was significantly inhibited with *L. plantarum* at 10^8^ CFU/mL in the multi-species model. These results were consistent with other research, which found that acidic pH repressed the yeast-to-filamentous transition [50]. In addition, *ERG4* and *CHT2* were only significantly upregulated in the 10^7^ CFU/mL of *L. plantarum* multi-species groups. *ERG4* encoded sterolC-24 reductase and was related to antifungal resistance. The *CHT2* gene encoded the chitinase 2 precursor related to yeast cell wall chitin remodeling [51]. The upregulation of *ERG4* and *CHT2* genes resulted in increasing resistance to stress and surviving in the competitive interactions for *C. albicans*. This may explain why *C. albicans* can thrive with *L. plantarum* at 10^8^ CFU/mL in the multi-species model.

The genes associated with the virulence of *S. mutans* were also altered in the dual- and multi-species models containing *L. plantarum*. The *atpD* gene was upregulated with *L. plantarum* at 10^8^ CFU/mL in the multi-species group, compared to the dual-species control group (*C. albicans* + *S. mutans*) at 20 h. This was consistent with the previous study [27]; a probable explanation is that *S. mutans* cells may use most of their energy to maintain internal pH homeostasis under acidic circumstances. This physiological process was associated with the *atpD* gene [52]. The acidogenic properties of *S. mutans* were inhibited in the presence of 10^8^ and 10^7^ CFU/mL of *L. plantarum*, as indicated by the downregulation of the *lacC* and *lacG* genes.

With regard to the research methods, some limitations need to be acknowledged. Firstly, a limitation of this study is that a planktonic model was employed. Biofilm and animal models, together with potential clinic studies, need to be carried out to assess the effect of *L. plantarum* on caries prevention. Secondly, our study introduced glucose as the sugar challenge in the planktonic model, so future biofilm and animal studies should assess the condition when other types of carbohydrates such as sucrose are tested in the multi-species interaction. Thirdly, qRT-PCR was employed as a critical methodology in this study. However, it should be noted that RNA sequencing might offer more precision in data acquisition and analysis.

## 5. Conclusions

In summary, *L. plantarum* demonstrated dose-dependent antifungal and antibacterial effects against *C. albicans* and *S. mutans*. *L. plantarum* emerged as a promising candidate for the creation of novel antimicrobial probiotic products targeting dental caries prevention. Further research is warranted to identify the functional metabolites produced by *L. plantarum* at different dosages when interacting with *C. albicans* and *S. mutans*.

## Data Availability

All data generated or analyzed during this study are included in this article. Further enquiries can be directed to the corresponding author.

## Supplementary Materials

The following supporting information can be downloaded at https://www.mdpi.com/article/10.3390/pathogens12060848/s1. Figure S1: Schematic study design; Figure S2: Changes in species composition in dual species; Figure S3: pH in culture media; Figure S4: Effect of *L. plantarum* on the expression of *C. albicans* and *S. mutans* genes in multi-species model at six hours; Figure S5: Dose-related expression of *L. plantarum* gene in multi-species model at twenty hours; Figure S6: Dose-dependent inhibition of *C. albicans* hyphae formation by *L. plantarum* gene in multi-species model at ×20 magnification; Table S1: Gene of interest; Table S2: Primers used in qRT-PCR.
