# Supplementary material for: Dose-Dependent Inhibitory Effect of Probiotic Lactobacillus plantarum on Streptococcus mutans-Candida albicans Cross-Kingdom Microorganisms
Source: Pathogens. 2023 Jun 20;12(6):848. doi: 10.3390/pathogens12060848 (PMC10301334; doi:10.3390/pathogens12060848)
Supplement: Supplementary file 1 [file pathogens-12-00848-s001.zip › pathogens-2416299-supplementary.pdf]

Supplement

Table S1. Genes of interest

Microorganisms	Genes	Function
	plnA	Encode plantaricins
L. plantarum	*plnN*	Encode plantaricins
	PcrA	ATP-dependent DNA helicase
	atpD	ATPase complex and acid tolerance
S. mutans	*eno*	Putative enolase
	lacC/G	Galactose metabolism
	HWP1	Hyphal growth and adhesion to host cells
C. albicans	*ECE1*	Hyphal growth and adhesion to host cells
	CHT2	Fungal cell wall chitin remodeling
	ERG4	Antifungal medication resistance

Table S2. Primers used in RT-qPCR [27]

Genes	Primers	Sequence	Amplicon size (bp)
atpD	Sm_atpD_F	TGTTGATGGTCTGGGTGAAA	176
	Sm_atpD_R	TTTGACGGTCTCCGATAACC	
eno	Sm_eno_F	CAGCGTCTTCAGTTCCATCA	194
	Sm_eno_R	TCACTCAGATGCTCCAATCG	
lacG	Sm_lacG_F	ATTGGATGCGTGCTTTTGATGG	94
	Sm_lacG_R	CGACCGACACCCTTAATCTGG	
lacC	Sm_lacC_F	GCTGGAATTACATCGGCTCTTGC	157
	Sm_lacC_R	CCTCCGCTACCTCAATTTGTTGG	
ACT1	Ca_ACT1_F	TGCTCCAGAAGAACACCCA	182
	Ca_ACT1_R	CACCTGAATCCAAAACAATACCAGT	
HWP1	Ca_HWP1_F	TGGTGCTATTACTATTCCGG	182
	Ca_HWP1_R	CAATAATAGCAGCACCGAAG	
ECE1	Ca_ECE1_F	GCTGGTATCATTGCTGATAT	168
	Ca_ECE1_R	TTCGATGGATTGTTGAACAC	
CHT2	Ca_CHT2_F	TTGGGATGCTTCTGGGGCTT	111
	Ca_CHT2_R	GCAGAAGAAGATGGGGCAACAC	
ERG4	Ca_ERG4_F	TCAAATGTGCCAATGGTTCT	101
	Ca_ERG4_R	AGCCCAAGTCAATGTTTGAA	
rpoB	Lp14_rpoB_F	CACCGTACCCGTAGAAGTTATGC	106
	Lp14_rpoB_R	GGAGACCTTGATCCAAGAACCA	
pcrA	Lp14_pcrA_F	AGGAGGTCTGGGTCTCAACG	118
	Lp14_pcrA_R	AAGGTCCGTTGCTCGCTAGT	
plnN	Lp14_plnN_F	ATTGCCGGGTTAGGTATCG	146
	Lp14_plnN_R	CCTAAACCATGCCATGCAC	
plnA	Lp14_plnA_F	GTGGAAAGAGTAGTGCGTATTC	135
	Lp14_plnA_R	CGCCATCTATACGAAATATAACTTG	

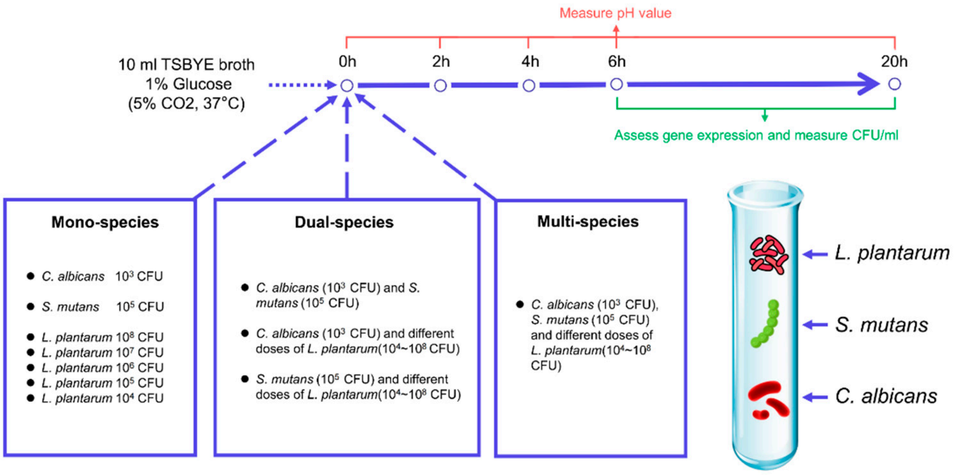

Figure S1. Schematic study design

The starting concentration for microorganisms was 10^3 CFU/ml for *C. albicans*, 10^5 CFU/ml for *S. mutans* and 10^4-10^8 CFU/ml for *L. plantarum*. *C. albicans* (10^3 CFU/ml) and *S. mutans* (10^5 CFU/ml) were used to mimic high caries risk in a clinical condition. The maximum inoculation level of *L. plantarum* (10^8 CFU/ml) is the lower dose of probiotics utilized in commercial probiotic products (10^9–10^{12} CFU/ml as a single dosage). Mono-species, dual-species and multi-species model were used to assess the intervention between *C. albicans*, *S. mutans* and different doses of *L. plantarum* (10^4 – 10^8 CFU/ml).

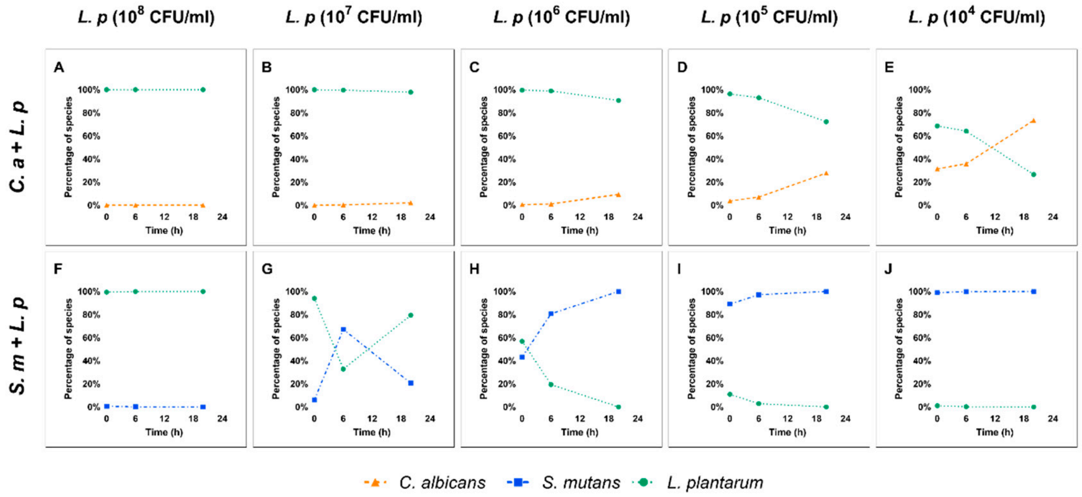

Figure. S2. Changes of species composition in dual-species

The composition of each microorganism in dual-species were showed. (A–E) The composition of *C. albicans* and *L. plantarum* in dual-species condition. (F–J) The composition of *S. mutans* and *L. plantarum* in dual-species condition.

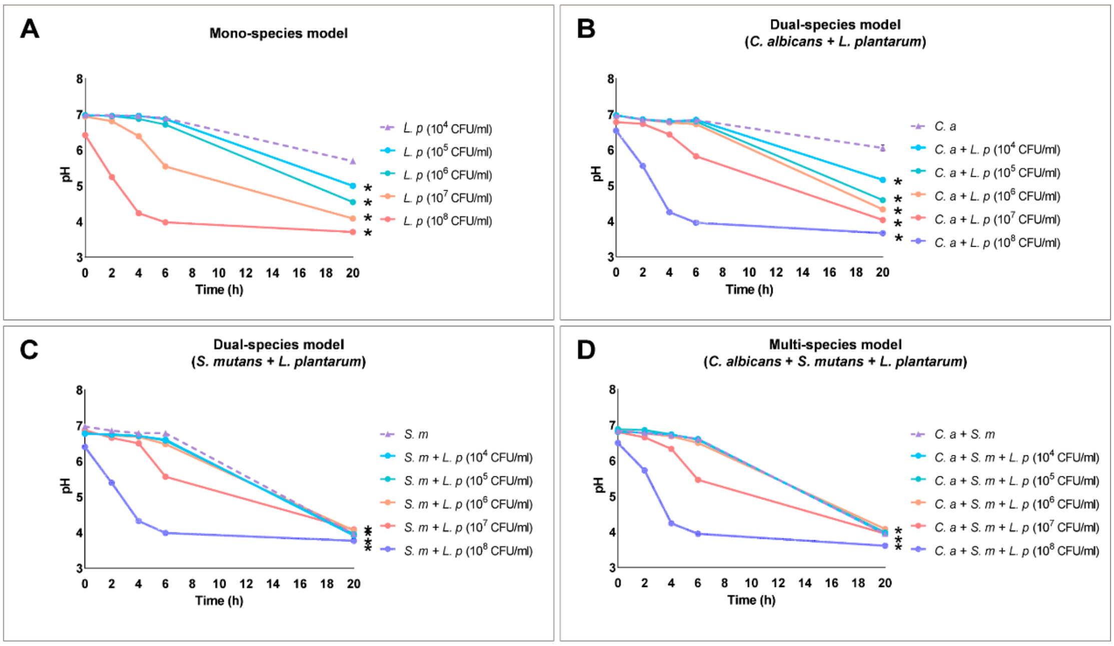

Figure. S3. pH in culture media

(A) pH in mono-species condition. (B) pH in *C. albicans* presence dual-species condition. (C) pH in *S. mutans* presence dual-species condition. (D) pH in multi-species condition. pH decreased faster and to lower value by adding a high concentration of 10^8 CFU/ml *L. plantarum.* The dotted line represents the control groups in each model. * Indicates the pH values of other groups were significantly different from the pH values of the control group at 20 h ($p < 0.05$).

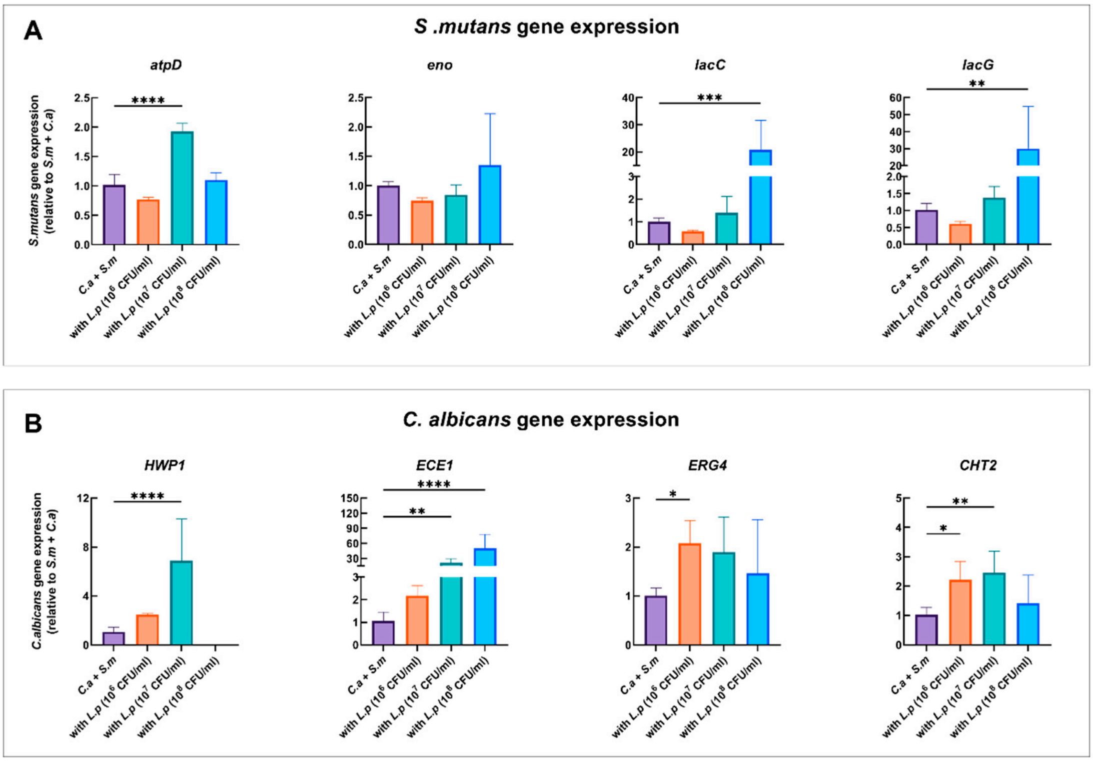

Figure. S4. Effect of *L. plantarum* on the expression of *C. albicans* and *S. mutans* genes *in* multi-species model at six hours

qRT-PCR was performed for *S. mutans* and *C. albicans* genes of interest for mixed-species culture at 6 h. *S. mutans* (A) and *C. albicans* (B) gene expression ratio was shown, the comparison was relative to *S. mutans* and *C. albicans* dual-species. p values were determined by one-way ANOVA with post hoc test. *$p < 0.05$, **$p < 0.01$, ***$p < 0.001$, ****$p < 0.0001$.

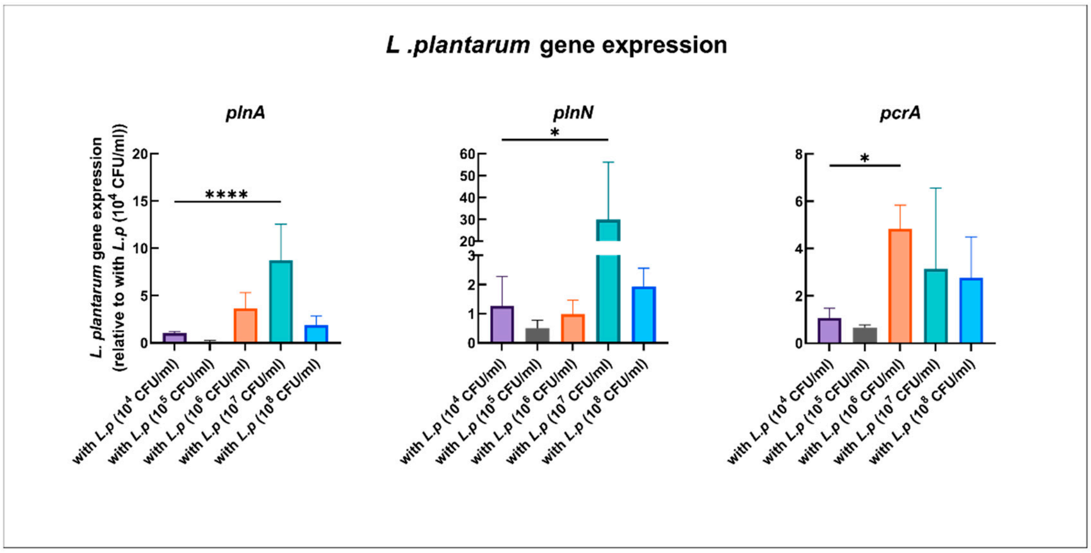

Figure. S5. Dose-related expression of *L. plantarum* gene *in* multi-species model at twenty hours

qRT-PCR was performed for *L. plantarum* genes of interest for mixed-species at 20 h. *L. plantarum* gene expression ratio was shown, the comparison was relative to 10^4 CFU/ml *L. plantarum* multi-species group. p values were determined by one-way ANOVA with post hoc test. **p* <0.05, ***p* <0.01, ****p* <0.001, *****p* <0.0001.

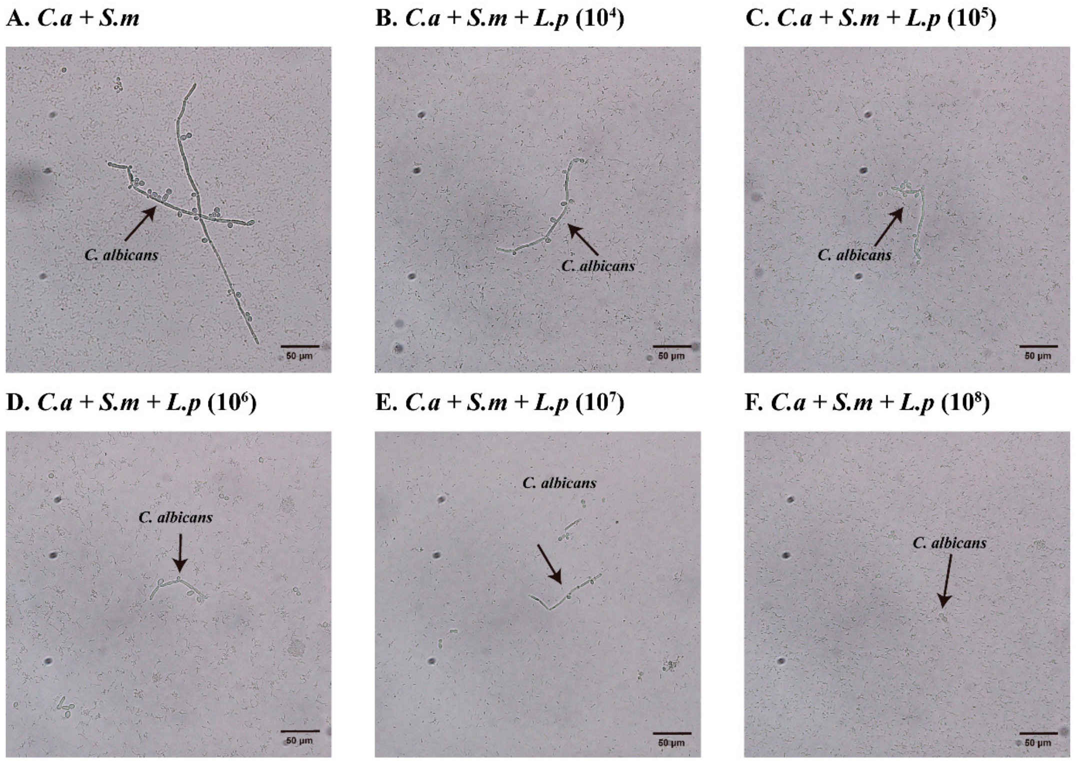

Figure S6: Dose-dependent inhibition of C. albicans hyphae formation by L. plantarum gene in multi-species model at ×20 magnification (A–F)
